# Effect of a self-assembled 3D flower-like hierarchical architecture on the thermoelectric properties of Ag-doped Bi_2_S_3_†

**DOI:** 10.1039/d4ra04467c

**Published:** 2024-11-28

**Authors:** T. Manimozhi, S. Kavirajan, M. Navaneethan, A. Joseph Sagaya Kennedy

**Affiliations:** a Department of Physics, Saveetha School of Engineering, Saveetha Institute of Medical and Technical Sciences (SIMATS), Saveetha University Thandalam Chennai-602105 India manimozhiavc@gmail.com; b Nanotechnology Research Centre (NRC), SRM Institute of Science and Technology Kattankulathur 603203 Tamil Nadu India m.navaneethan@gmail.com; c Functional Materials and Energy Devices Laboratory, Department of Physics and Nanotechnology, SRM Institute of Science and Technology Kattankulathur 603203 Tamil Nadu India; d Department of Physics, SRM TRP Engineering College (SRM Group) Irungalur Tiruchirappalli-621105 Tamil Nadu India

## Abstract

Powder samples of Bi_2_S_3_ and Ag-doped Bi_2_S_3_ compounds were successfully synthesized *via* the solvothermal method. The synthesized powders were consolidated using the cold-press method and annealed at 300 °C for 3 h. The cross-sections of the consolidated samples exhibited a densely packed hierarchical architecture micro-flower-like morphology. The effect of Ag doping on the thermoelectric properties of the samples was systematically studied. The results showed that Ag doping had considerable effects on the morphology, leading to grain boundary scattering and point defects. The addition of 0.025% Ag led to an 81% enhancement in electrical conductivity (*σ*) at 550 K. However, a reduction in the Seebeck coefficient (*S*) was observed, and the power factor (*S*^2^*σ*) was enhanced. Owing to the scattering of all-scale phonons caused by the hierarchical architecture, a low thermal conductivity of 0.407 W m^−1^ K^−1^ at 483 K was obtained, which is one of the low values among the reported Bi_2_S_3_ materials. The maximum *zT* was obtained as 0.06 at 543 K for the 0.025% Ag-doped Bi_2_S_3_ sample.

## Abstract 2

Ag doping in Bi_2_S_3_ improved thermoelectric properties by lowering the bandgap, enhancing conductivity, and reducing thermal conductivity to 0.415 W mK^−1^*via* phonon scattering. At 543 K, 0.025% Ag-doped Bi_2_S_3_ achieved a power factor of 50.1 μW mK^−2^.

## Graphical abstract

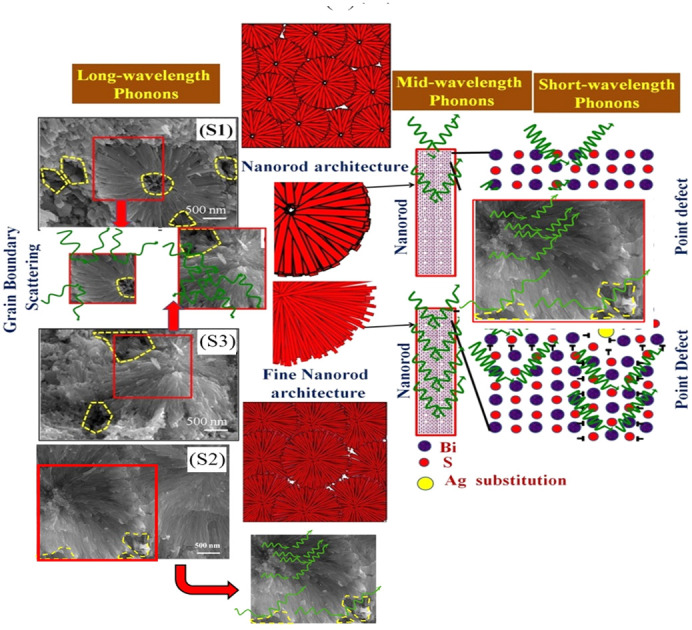

## 1. Introduction

Over the past few decades, rapid progress in developing numerous thermoelectric materials has been witnessed for waste heat recovery and solid-state cooling. Thermoelectric materials have recently triggered the demonstration of excellent performance in energy conversion, without releasing any hazardous by-products in the environment. The energy efficiency of thermoelectric materials is determined using the dimensionless figure of merit (*zT*), which is defined as *zT* = (*σS*^2^*T*)/(*κ*_e_ + *κ*_l_), where *σ*, *S*, *T*, *κ*_e_ and *κ*_l_ denote electrical conductivity (S m^−1^), Seebeck coefficient (μV K^−1^), absolute temperature (K), and electronic and thermal conductivity of the lattice (W m^−1^ K^−1^), respectively.^1^ The *zT* value has been enhanced using many strategies. In general, the reduction of lattice thermal conductivity is achieved through all scale hierarchical architecturing^2^ and grain boundary phonon scattering.^3^ Optimization of the power factor has been accomplished through doping,^4–6^ band structure convergence,^7^ quantum confinement,^8^ formation of resonant states,^9^ carrier energy filtering^10^ and minority carrier blocking.^11^

Thermoelectricity (TE) has witnessed major achievements over the years; however, it is still a major challenge to realize synergistic optimization of lattice thermal conductivity and the power factor because of the strong coupling between these fundamental parameters. Excellent TE characteristics are displayed by materials based on Bi_2_Te_3_ (ref. 12 and 13) and PbTe^14,15^ at room temperature and medium temperature, respectively. Their cost-prohibitive and highly toxic compositions, however, restrict their potential large-scale usage. Owing to its high Seebeck coefficient and low lattice thermal conductivity, bismuth sulphide (Bi_2_S_3_) is a thermoelectric compound with significant research value at moderate temperatures. In contrast to the related compound Bi_2_Te_3_, it is less toxic, more affordable, and more environmentally friendly. Bi_2_S_3_, however, has very poor electrical conductivity, which limits its commercial use.

Bi_2_S_3_ is a good semiconducting material with a direct bandgap of 1.3 eV. To date, enormous efforts have been devoted to the structural modification of Bi_2_S_3_ to enhance its thermoelectric properties. For example, the electrical resistivity of Bi_2_S_3_ was reduced by 3 orders of magnitude by introducing sulfur vacancies in the lattice positions.^16^ Cui *et al.*^17^ reported a reduction of the thermal conductivity together with a high *zT* value for Ag-doped in a Bi–Te alloy, which showed a 30% thermoelectric improvement compared with the undoped alloy. Also, the doping of Ag in the Bi sites in Bi_2_Se_3_ enhanced the *zT* value due to the increment in the mobility and carrier concentration of free electrons. In this context, Ag has a 5s^1^ electron configuration in the outer orbital while Bi has a 6p^3^ electron. Bi_2_S_3_ and Bi_2_Se_3_ have similar structures. Hence, the doping of Ag in Bi–S based alloys could be expected to increase the structural defects. Recently, suitable element doping and a hierarchically organized microstructure morphology have been employed to enhance the *zT* value and low thermal conductivity of thermoelectric materials. For example, Sun *et al.*^18^ reported that doping Mn^2+^ in the Sb^3+^ sublattice increased the carrier concentration, together with a reduction in lattice thermal conductivity, which resulted in a high *zT* value (AgSb_0.96_Mn_0.04_Se_2_) of ∼1.05 at 673 K. Wang *et al.*^19^ reported that Ag doping and embedding a Pt quantum dot in Mn_0.990_Ag_0.010_Si_1.8_ led to it exhibiting a higher *zT* value (52% increase at 823 K) due to decoupling the modulation between the electrical and thermal transport properties. Zhou *et al.*^20^ reported self-assembled 3D flower-like hierarchical Ti-doped Cu_3_SbSe_4_ microspheres that demonstrated a low lattice thermal conductivity of 0.38 W m^−1^ K^−1^ at 623 K (Cu_3_Sb_0.93_Ti_0.07_Se_4_) and a high *zT* value of ∼0.59 at 623 K (Cu_3_Sb_0.96_Ti_0.04_Se_4_). All-scale hierarchical architectures represent a potential strategy to significantly decrease *κ*_total_ to reach the minimum theoretical limit,^21^ but it is important that the power factor is not appreciably compromised.^22^ Hence, the intellectual challenge for next-generation thermoelectric materials is to synthesize hierarchically organized microstructures^20^ without compromising the power factor.

Herein, we prepared hierarchically organized architectures of Bi_2_S_3_ and Ag-doped Bi_2_S_3_ by a solvothermal method. The fundamental and functional properties of the prepared compounds were studied by various analytical techniques. It was found that the hierarchical structure could reduce the thermal conductivity. The electrical conductivity was enhanced due to the Ag doping. Hence, Ag-doped Bi_2_S_3_ provides an alternative pathway to develop toxic-free thermoelectric materials with high performance.

## 2. Experimental section

### 2.1 Synthesis procedure

All the chemicals were analytical-grade reagents and were purchased from SISCO Research Laboratories Pvt. Ltd. (SRL), India. Bismuth(iii) nitrate pentahydrate (Bi(NO_3_)_3_·5H_2_O), polyethylene glycol (PEG, MW = 20 000), ethylene glycol (C_2_H_6_O_2_ – EG), and thiourea (CH_4_N_2_S) were used as reagents without further purification. In accordance with the synthesis procedures outlined by Manimozhi *et al.*^23^ and Dechong *et al.*,^24^ we utilized PEG in our method for both size control and growth regulation. These references support our approach for optimizing the synthesis process. First, 0.01 mol of Bi(NO_3_)_3_·5H_2_O and 2 g of PEG were dissolved in 30 mL EG, and the solution was left to stand at room temperature for 3 h without disturbance. Afterwards, 0.02 mol of thiourea was added to the above solution and stirred for a few minutes until uniform dissolution of the precursor was observed. The precursor was then hydrothermally treated in a Teflon-lined autoclave at 180 °C for 20 h. Next, centrifugation was performed using DI water and ethanol. Finally, black precipitates were obtained following drying at 80 °C, and the sample was denoted as S1. Similarly, 0.0002 mol (0.01%) and 0.0005 mol (0.025%) of Ag(NO_3_) was added with 0.0098 mol and 0.0095 mol of Bi(NO_3_)_3_·5H_2_O, PEG to prepare the Ag-substituted samples, which were denoted as S2 and S3.

### 2.2 Characterization

The crystalline phases and structures were examined by XRD using a Malvern PANalytical, MAERIS high-resolution benchtop X-ray diffraction system with CuK_α_ radiation (*λ* = 1.5406 Å) at a scanning speed of 0.18° per minute and in the scanning angle range from 10° to 60°. The optical reflectance of the samples was obtained from the UV-Vis spectra using a 3600 PLUS: UV-Vis-NIR spectrometer equipped with an integrating sphere. BaSO_4_ was used as the 100% reflectance standard. X-Ray photoelectron spectroscopy (XPS) was also performed using a ULVAC-PHI, Inc. spectrometer (PH15000), with an Al-K_α_ radiation source with an excitation energy of 1486.6 eV. Cross-sectional micrographs and their elemental mappings were obtained by EDS analysis of pellet samples using a high-resolution scanning electron microscopy (HR-SEM) system.

### 2.3 Thermoelectric measurements

The powder samples were pressed into pellets using a metal die of 13 mm diameter under 50 MPa pressure. The relative density of the samples was ∼81% of the theoretical density (6.78 g cm^−3^) of Bi_2_S_3_.^25^ The samples were annealed at 300 °C in a tubular furnace in an Ar gas atmosphere for 3 h and then naturally cooled to room temperature. The total thermal conductivity (*κ*_total_) of the bulk samples was measured from the relation: *κ*_total_ = *DC*_p_*ρ*, where *D* is the thermal diffusivity, *ρ* is the density, and *C*_p_ is the specific heat capacity of the sample. The specific heat capacity (*C*_p_) was indirectly taken using Pyroceram 9606 as a reference sample in the temperature range from 303 K to 543 K and the thermal diffusivity (*D*) measurements were carried out using the laser flash method (Netzsch, LFA467HT). The density of the samples (*ρ*) was measured by Archimedes' method. The Seebeck coefficient and electrical resistivity were measured using a Seebeck coefficient/electric resistance measuring system (ZEM-3, Advance RIKO) under a He atmosphere.

## 3. Results and discussion

### 3.1 Phase structural analysis by XRD and XPS

**Fig. 1:**
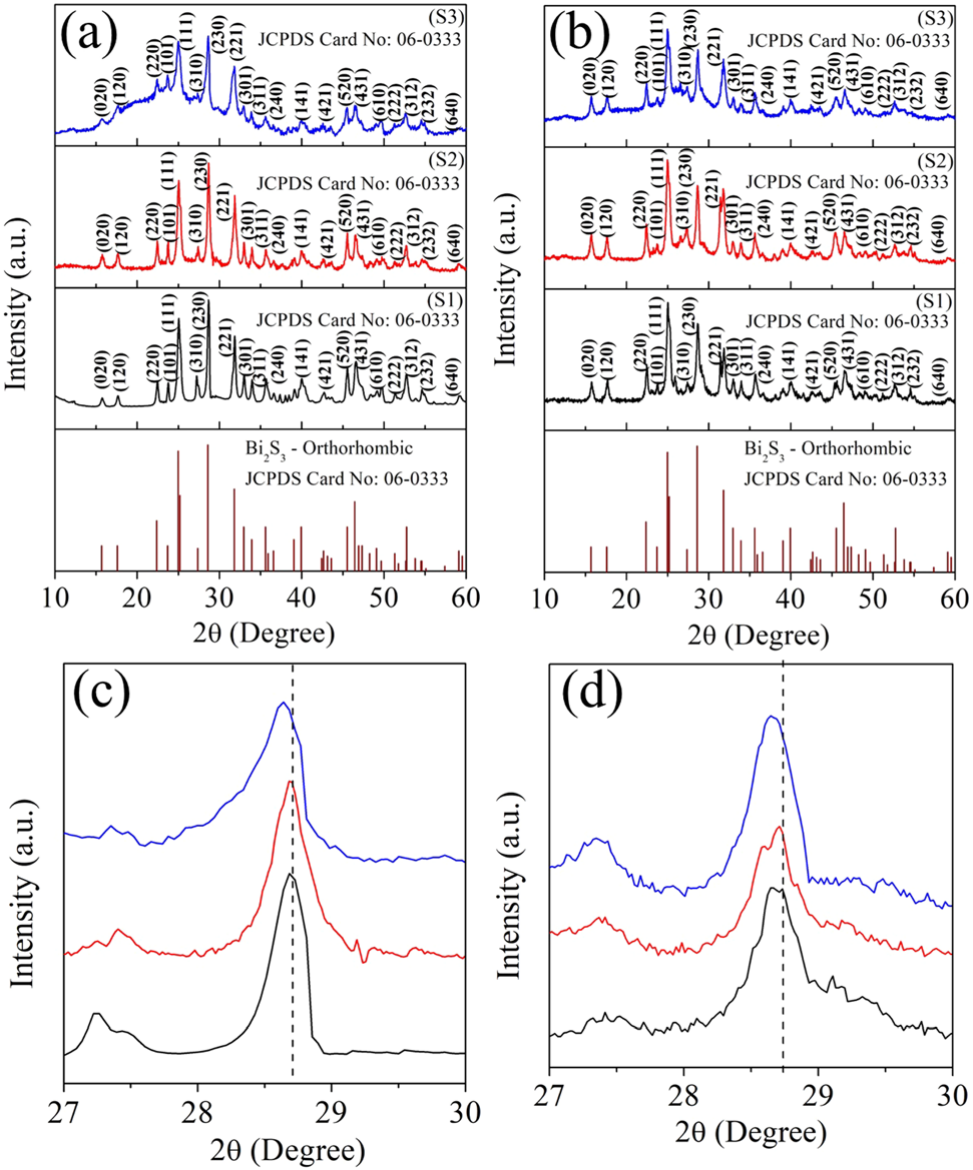
XRD patterns of the samples S1 (Bi_2_S_3_), S2 (0.01% Ag-doped Bi_2_S_3_), and S3 (0.025% Ag-doped Bi_2_S_3_) (a and c) for the as-synthesized powders; (b and d) for pellets.

Fig. 1a shows the XRD patterns of the powder samples while Fig. 1b shows the XRD patterns of the cold-pressed pellet samples, exhibiting the formation of the orthorhombic phase of Bi_2_S_3_, which well-matched the standard JCPDS card no: 06-0333. Compared to the pure sample (S1), the peak intensity ratio of the dominant planes of the 0.025% Ag-substituted sample (S3) was decreased and slightly shifted to the lower angle side. This was due to the doping of Ag ions into the Bi_2_S_3_ matrix, which affected the growth orientation. In the case of the other Ag-doped sample (S2), there was a slight peak shift to the lower angle side without disturbing the peak intensity. For example, the diffraction peak for the (2 3 0) plane appeared at 28.68°, and it was shifted to a lower diffraction angle due to the incorporation of the larger ionic radius Ag ion (Ag^+^ = 115 pm) in the Bi^3+^ (96 pm) matrix. Fig. 1c and d display the corresponding (2 3 0) peak with a noticeable lower angle shift, indicating the enlargement of the unit cell size. The unit cell parameters were calculated from the diffraction peaks, and the cell parameters are listed in Table 1, which confirmed the slight enlargement in the cell parameters value for the S2 sample compared to the pure sample S1.

**Table 1:** Estimated values of lattice parameters for the powder samples S1, S2 and S3

Sample code	Lattice parameters		
	*a* (Å)	*b* (Å)	*c* (Å)
S1 (powder)	11.160	11.249	3.976
S2 (powder)	11.160	11.253	3.978
S3 (powder)	11.160	11.261	3.978
S1 (bulk)	11.129	11.241	3.978
S2 (bulk)	11.232	11.248	3.980
S3 (bulk)	11.299	11.257	3.987

**Fig. 2:**
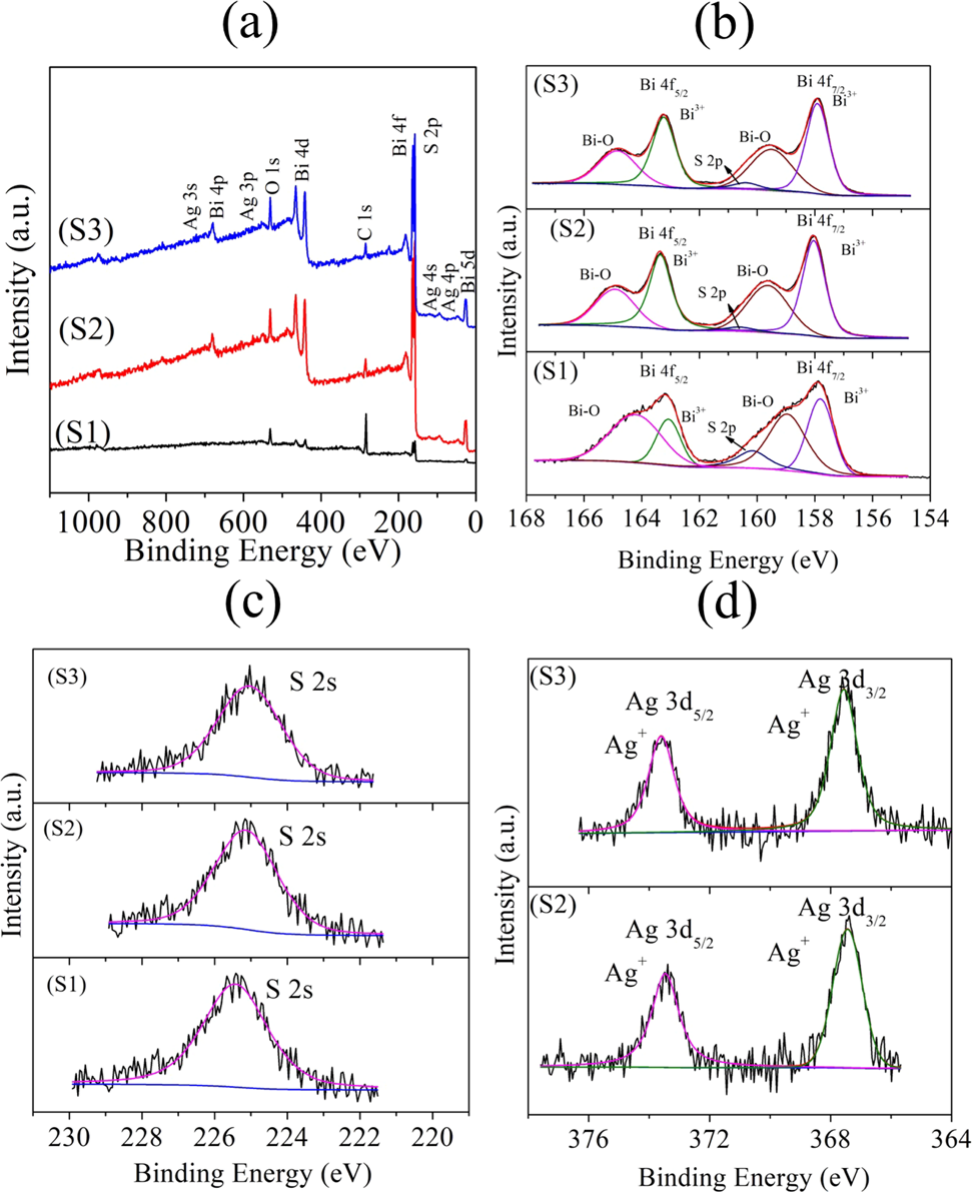
XPS spectra of the samples S1 (Bi_2_S_3_), S2 (0.01% Ag-doped Bi_2_S_3_), and S3 (0.025% Ag-doped Bi_2_S_3_): (a) survey spectra; core level spectra of (b) Bi 4f, (c) S 2s, and (d) Ag 3d.

X-Ray photoelectron spectroscopy (XPS) was performed to elucidate the chemical states of the elements in Bi_2_S_3_ (S1) and the 0.01% and 0.025% Ag-doped Bi_2_S_3_ (S2) and (S3) samples. The survey spectra of the samples suggested the presence of Bi, Ag, S, C, and O (Fig. 2a). In the core level Bi 4f spectra (Fig. 2b), peaks were observed at 157.80 and 163.06 eV for S1; 158.85 and 163.10 eV for S2; and 157.90 and 163.20 eV for S3 due to the spin–orbit splitting of Bi 4f_5/2_ and Bi 4f_7/2_, respectively, confirming the Bi^3+^ oxidation state.^26^ The interaction of the surface oxygen with the sample (Bi–O) was also observed by the peaks at the higher binding energy side. Additionally, peaks were obtained at 160.15 eV (S1), 160.28 eV (S2), and 160.37 eV (S3) correlated to the binding energies of S 2p.^26^Fig. 2c shows the binding energies of S 2 s with peaks located at 225.60 eV for S1, 225.16 eV for S2, and 225.08 eV for S3.^27^ In the core level spectra of Ag 3d (Fig. 2d), peaks appeared at binding energies of 367.54 and 373.65 eV for S2; and at 367.4 and 373.4 eV for S3. These peaks were associated with Ag 3d_5/2_ and Ag 3d_3/2_, confirming the oxidation state of Ag^+^.^27^ The XPS results suggested the occurrence of a chemical shift by the changes in the corresponding binding energies for Bi 4f in S 2s and S 2p, respectively. This was due to the lower electronegativity of Ag (1.93, according to Pauling's scale) than Bi (2.02), which thus modified the electron density around the Bi ions.

### 3.2 Optical bandgap by UV-Vis-NIR analysis

**Fig. 3:**
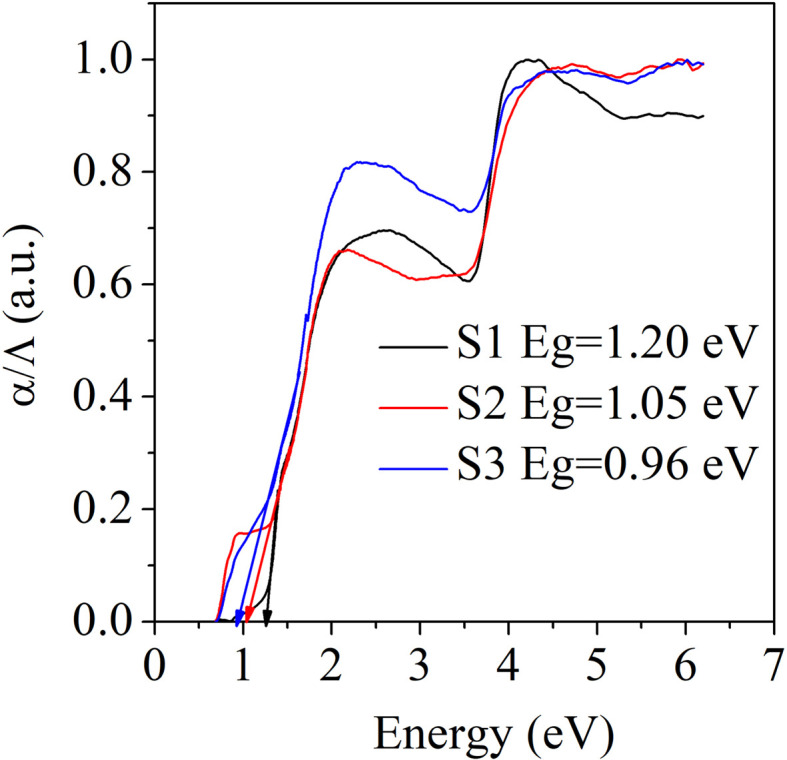
Optical bandgaps of the samples S1 (Bi_2_S_3_), S2 (0.01% Ag-doped Bi_2_S_3_), and S3 (0.025% Ag-doped Bi_2_S_3_).

Fig. 3 presents the optical bandgaps determined for the S1, S2, and S3 samples from the UV-Vis absorption spectra. These were calculated using Kubelka–Munk's equation,^28^*α*/*Λ* = (1 − *R*)^2^/(2*R*), where *R* is the reflectance, *α* is the absorption and *Λ* is the scattering coefficient, respectively. The optical bandgaps of the samples were at 1.20 eV for S1 1.05 eV for S2, and 0.96 eV for S3. The bandgap of the Bi_2_S_3_ sample (S1) matched with the reported value of 1.29 eV.^29^ The decrement in the optical band gaps of samples S2 and S3 was due to the Ag doping, which may have introduced an impurity level in Bi_2_S_3_.

### 3.3 Morphological analysis by HR-SEM

**Fig. 4:**
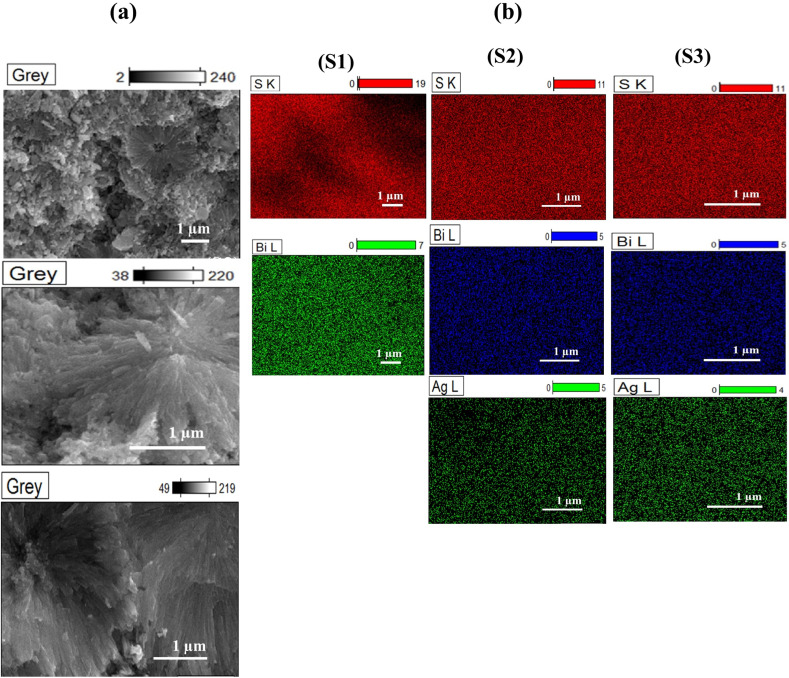
(a) HR-SEM micrographs and (b) elemental mapping for cross-sectional views of the pellet samples S1 (Bi_2_S_3_), S2 (0.01% Ag-doped Bi_2_S_3_), and S3 (0.025% Ag-doped Bi_2_S_3_).

Fig. 4 shows the HR-SEM micrographs and elemental mapping of the fractured surfaces of the cold-pressed bulk samples. The samples had organized hierarchical microstructures with pores. The number of pores in S1 was higher than in S2 and S3. It was observed that Ag doping suppressed the growth of the internal pores, as shown in Fig. 4a. The elemental mapping of the cross-sectional pellet samples clearly showed the uniformly distributed elements of Bi and S for the S1 sample as well as the distributions of Bi, S, and Ag for the S2 and S3 samples (Fig. 4b). The elemental compositions and spectra of the Bi_2_S_3_ (S1), 0.01% Ag-doped Bi_2_S_3_ (S2), and 0.025% Ag-doped Bi_2_S_3_ (S3) samples based on EDS analysis are outlined in Table ES1 and Fig. ES1.†

### 3.4 Thermoelectric properties

**Fig. 5:**
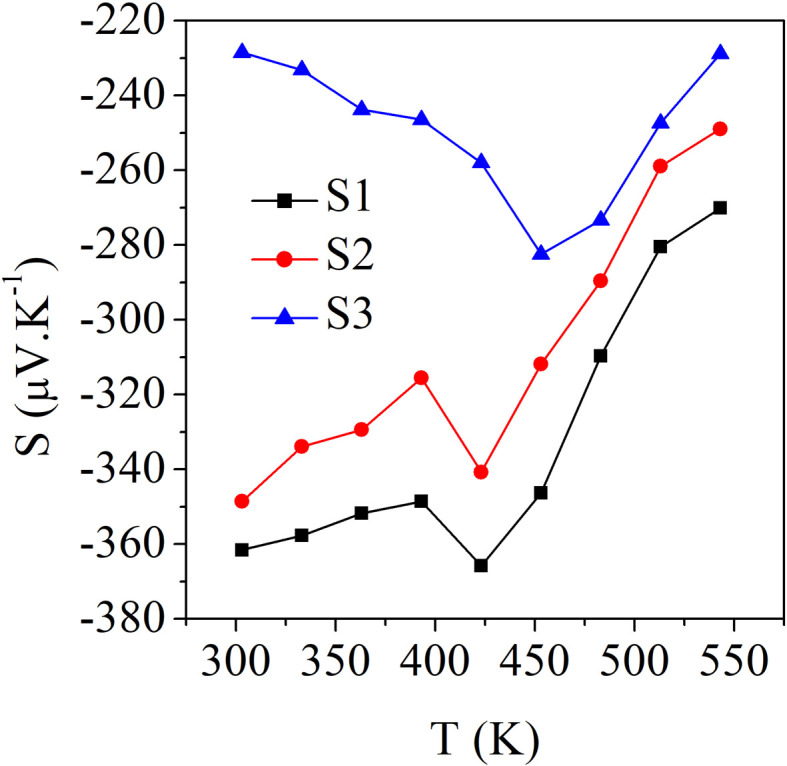
Seebeck coefficient as a function of temperature for the samples S1 (Bi_2_S_3_), S2 (0.01% Ag-doped Bi_2_S_3_), and S3 (0.025% Ag-doped Bi_2_S_3_).

Fig. 5a shows the experimental data for the Seebeck coefficient as a function of the temperature, for which negative values were observed, indicating that the majority of the charge carriers were electrons (n-type behaviour). For the sample S1 (Bi_2_S_3_), the values decreased from 362 μV K^−1^ at 303 K to 270 μV K^−1^ at 543 K. In the case of sample S2, the values decreased from 348 μV K^−1^ at 303 K to 246 μV K^−1^ at 543 K. In contrast, for sample S3, the room temperature value (228 μV K^−1^ at 303 K) increased to 282 μV K^−1^ at 453 K and then decreased back to 228 μV K^−1^ at 543 K. The maximum Seebeck coefficient was obtained as 366 μV K^−1^ at 423 K for the S1 sample. Generally, the Seebeck coefficient is directly proportional to the atomic scattering factor and inversely proportional to the carrier concentration.^30^ The increase–decrease phenomenon of the Seebeck coefficient was attributed to minor thermal and structural fluctuations in the samples, as indicated by the TG-DTA analysis (Fig. ES2†), which showed there were no significant structural changes. These fluctuations were observed at 423 K for S1 (Bi_2_S_3_) and at 453 K for both S2 (0.01% Ag-doped Bi_2_S_3_) and S3 (0.025% Ag-doped Bi_2_S_3_). This behaviour may be linked to lattice distortions or a slight thermal expansion.^31^

**Fig. 6:**
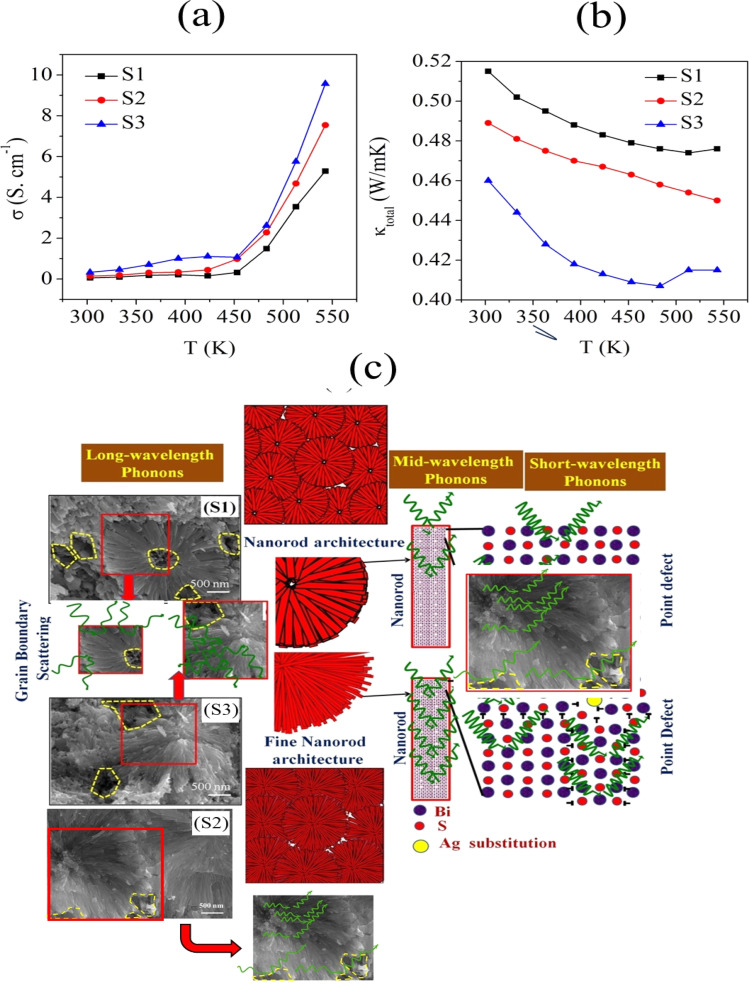
(a) Temperature-dependent electrical conductivity, and (b) temperature-dependent total thermal conductivity of the samples S1 (Bi_2_S_3_), S2 (0.01% Ag-doped Bi_2_S_3_), and S3 (0.025% Ag-doped Bi_2_S_3_). (c) Schematic of the phonon scattering mechanism of the hierarchical architectures of Bi_2_S_3_ (S1), 0.01% Ag-doped Bi_2_S_3_ (S2), and 0.025% Ag-doped Bi_2_S_3_ (S3).

Fig. 6a shows the electrical conductivity (*σ*) plots of the S1, S2, and S3 samples with the temperature ranging from 300 to 550 K, showing *σ* increased from 0.054 to 5.287 S cm^−1^, 0.138 to 7.537 S cm^−1^, and 0.325 to 9.571 S cm^−1^ for samples S1, S2, and S3, respectively. This tendency for an increase in electrical conductivity with increasing temperature was due to the semiconductor behaviour.^32^ Sample S3 (0.025% Ag-doped Bi_2_S_3_) showed enhanced electrical conductivity compared to S1 and S2, due to the concentration of higher Ag-doping and the presence of a hierarchical structure and pores, which was consistent with reports.^33,34^ Also, the enhancement of the electrical conductivity was attributed to the reduced bandgap, which was consistent with the UV-Vis results. Furthermore, it was also attributed to the Ag doping, which affects the electronic structure and charge carrier density.

Generally, the electrical conductivity (*σ*) is directly proportional to the carrier concentration (*n*) and mobility (*μ*), and can be obtained from the expression *σ* = *neμ*. In samples S2 and S3, the addition of Ag (0.01% for S2 and 0.025% for S3) in interstitial sites of the Bi_2_S_3_ matrix led to an enhancement of its electrical conductivity. In n-type Ag-doped Bi_2_S_3_ materials, the electrons are majority carriers. Due to the increment in carrier concentration and decrement in mobility (Table 2), electrical conductivity enhancements were observed for the Ag-doped samples. A similar behaviour was also observed in a previous report.^35^

**Table 2:** Hall measurements for the carrier concentration and mobility of samples S1–S3

Sample code	Conduction	Carrier concentration (cm^−3^)	Mobility (cm^−2^ V^−1^ s^−1^)
S1	n-type	−1.09 × 10^18^	18
S2	n-type	−1.15 × 10^18^	9
S3	n-type	−1.21 × 10^18^	1.5

Fig. 6b shows the total thermal conductivity (*κ*_total_) as a function of temperature for the samples. At 303 K, the *κ*_total_ value was measured as 0.515 W m^−1^ K^−1^ for S1, 0.489 W m^−1^ K^−1^ for S2, and 0.46 W m^−1^ K^−1^ for S3. When increasing the temperature to 543 K, the values were reduced for the samples due to the strong phonon scattering, reducing to 0.476 W m^−1^ K^−1^ for S1, 0.45 W m^−1^ K^−1^ for S2, and 0.415 W m^−1^ K^−1^ for S3. The strong phonon scattering occurred due to the presence of a large number of grain boundaries and the edges of randomly distributed pores in the organized hierarchical microstructures. The present values of the thermal conductivity for samples S1, S2, and S3 showed low thermal conductivity values compared with the reported values, as indicated in Table 3.

**Table 3:** Comparison of the thermal conductivity in the present study with that reported in the literature

Materials	*κ*_total_ W m^−1^ K^−1^	Temperature (K)	Material preparation	Pellet preparation	Morphology	References
Bi_2_S_3_	∼0.77–0.59	303–624 K	Solid-state reaction	Spark plasma sintering	Grain sizes of 100–200 nm with dense structure	25
Bi_2_S_3_	0.74–0.56	303–573 K	Mechanical alloying	Spark plasma sintering	Dense microstructure with grain sizes of 200–300 nm	36
Bi_2_S_3_	∼0.62–0.42	303–657 K	Mechanical alloying	Spark plasma sintering	Average grain size of <500 nm	37
Bi_2_S_3_	∼0.83–0.48	723 K	Vacuum sealing	Spark plasma sintering	Lamellar structure with a layer stacking thickness of less than 5 μm	38
Ag-doped Bi_2_S_3_	∼0.95–0.52	300–750 K	Solid-state reaction similar methods	Spark plasma sintering	Dense structure with optimal grain sizes	39
Bi_2_S_3_	0.515–0.473	303–573 K	Solvothermal	Cold-press + annealing	Dense hierarchical architecture with porous nature	PW (S1)
0.01% Ag-doped Bi_2_S_3_	0.489–0.45	303–573 K	Solvothermal	Cold-press + annealing	Dense hierarchical architecture with porous nature	PW (S2)
0.025% Ag-doped Bi_2_S_3_	0.499–0.407	303–573 K	Solvothermal	Cold-press + annealing	Dense hierarchical architecture with porous nature	PW (S3)

Fig. 6c shows a schematic of phonon scattering in Bi_2_S_3_ (S1) 0.01% Ag-doped Bi_2_S_3_ (S2) and 0.025% Ag-doped Bi_2_S_3_ (S3). The greater number of grain boundaries, nanoscale, and point defects in the Ag-doped samples increased the number of phonon scattering centres, which reduced the thermal conductivity. Here, the Ag dopants can induce the mass contrast and lattice strain, which can significantly restrict the short-wavelength phonon transmission, leading to point defects at the atomic scale. The nanorods sized around 100 nm can be the scattering sources of medium-wavelength phonons. So, the total thermal conductivity of sample S3 was induced by the large degree of phonon scattering due to lattice vibration. The mesoscale grain boundaries were correlated to the scattering centres of long-wavelength phonons (typically >100 nm). Due to the scattering of the all-scale phonons, including (i) short-wavelength phonon scattering due to point defects (ii) mid-wavelength phonon scattering due to mesoscale nanorods (iii) long-wavelength phonon scattering due to grain boundaries, the total thermal conductivity was greatly reduced for the S3 sample.

**Fig. 7:**
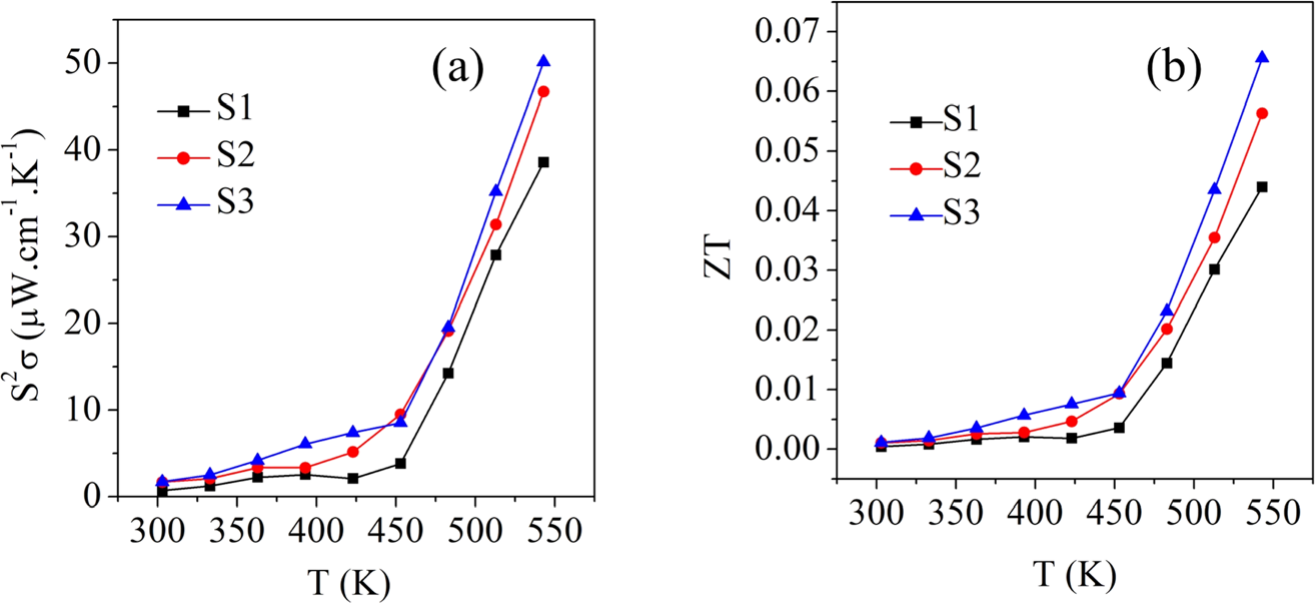
Temperature dependence of the (a) power factor and (b) *zT* values of the samples S1 (Bi_2_S_3_), S2 (0.01% Ag-doped Bi_2_S_3_), and S3 (0.025% Ag-doped Bi_2_S_3_).

Fig. 7a shows the temperature dependence of the power factor (*S*^2^*σ*) for the S1, S2, and S3 samples. The power factor of sample S1 was 0.7 μW cm^−1^ K^−2^ at 303 K and increased to 38.5 μW mK^−2^ at 543 K. For the S2 sample, it was 1.67 μW cm^−1^ K^−2^ at 303 K and increased to 46.6 μW mK^−2^ at 543 K. In contrast, S3 had a power factor of 1.7 μW cm^−1^ K^−2^ at 303 K, which increased to 50.1 μW mK^−2^ at 543 K. The trend of the power factor followed the trend of the electrical conductivity. This was due to the greater contribution of electrical conductivity compared to the Seebeck coefficient.

Fig. 7b shows the temperature-dependent figure-of-merit (*zT*) values for all the samples. It could be clearly seen that the *zT* value for the Bi_2_S_3_ sample (S1) increased from 0.0004–0.04 in the temperature range from 303 K to 543 K. With the addition of 0.01% Ag-doping, sample S2 showed *zT* values of 0.001–0.056 at 303–543 K, while the 0.025% Ag-doped sample S3 showed *zT* values of 0.001–0.06 at 303–543 K. Compared to the pure sample (S1), the 0.025% Ag-doped sample (S3) showed enhanced *zT* values with increasing the temperature due to its enhanced power factor (Fig. 7a) and low thermal conductivity (Fig. 6b).

## 4. Conclusion

In the present study, n-type Bi_2_S_3_ and Ag-doped Bi_2_S_3_ hierarchical nanostructures were synthesized by the solvothermal method and their thermoelectric properties were investigated. It was found that Ag doping had significant effects on the morphology and the thermoelectric properties of the doped Bi_2_S_3_. The bandgap of the Ag-doped Bi_2_S_3_ sample decreased whereas the electrical conductivity and power factor increased with increasing the Ag doping due to the increment in carrier concentration. Additionally, the thermal conductivity was reduced due to the intensive scattering of phonons by the all-scale hierarchical architecture. The mass fluctuations created by Ag doping increased the scattering of phonons, which resulted in the low thermal conductivity. In particular, the 0.025% Ag-doped Bi_2_S_3_ demonstrated a good thermoelectric performance, with a low thermal conductivity of 0.415 W m^−1^ K^−1^ and high-power factor of 50.1 μW mK^−2^ 543 K. Hence, the Ag doping of Bi_2_S_3_ along with ensuring a hierarchical architecture morphology is an effective approach to achieve a high thermoelectric performance.

## Data availability

All data underlying the results are available as part of the article, and no additional source data are required.

## Conflicts of interest

There are no conflicts to declare.

## Supplementary Material

RA-014-D4RA04467C-s001
