# Supplementary material for: Effect of a self-assembled 3D flower-like hierarchical architecture on the thermoelectric properties of Ag-doped Bi2S3
Source: RSC Adv. 2024 Nov 28;14(51):38035–43. doi: 10.1039/d4ra04467c (PMC11604178; doi:10.1039/d4ra04467c)
Supplement: RA-014-D4RA04467C-s001 [file RA-014-D4RA04467C-s001.pdf]

Supplementary Information (SI) for RSC Advances.
This journal is © The Royal Society of Chemistry 2024

Supplementary File

Self-assembled 3D Flower-like Hierarchical architecture on the thermoelectric properties of Ag-doped Bi_2S_3

T. Manimozhi[1*], S. Kavirajan[3], M. Navaneethan[2,3*], A. Joseph Sagaya Kennedy[4]

[1]*Department of Physics, Saveetha School of Engineering, Saveetha Institute of Medical and Technical Sciences (SIMATS), Saveetha University, Thandalam, Chennai -602105, India.*

[2]Nanotechnology Research Centre (NRC), SRM Institute of Science and Technology, Kattankulathur, 603203, Tamil Nadu, India.

[3]Functional Materials and Energy Devices Laboratory, Department of Physics and Nanotechnology, SRM Institute of Science and Technology, Kattankulathur-603203, Tamil Nadu, India.

[4]Department of physics, SRM TRP Engineering college (SRM Group), Irungalur, Tiruchirappalli - 621105, Tamilnadu, India.

***Corresponding Author**

E-mail address: manimozhiavc@gmail.com; m.navaneethan@gmail.com

Table ES1: Elemental composition of Bi_2S_3 (S1), 0.01 % of Ag-doped Bi_2S_3 (S2) and 0.025 % Ag-doped Bi_2S_3 (S3) samples based on EDS analysis.

Samples	Elements	Atom%	Weight%
**S1**	S	58.35	17.69
	Bi	41.65	82.31
**S2**	S	58.60	18.02
	Bi	40.36	80.90
	Ag	1.05	1.08
**S3**	S	53.55	15.39
	Bi	43.78	82.02
	Ag	2.67	2.58

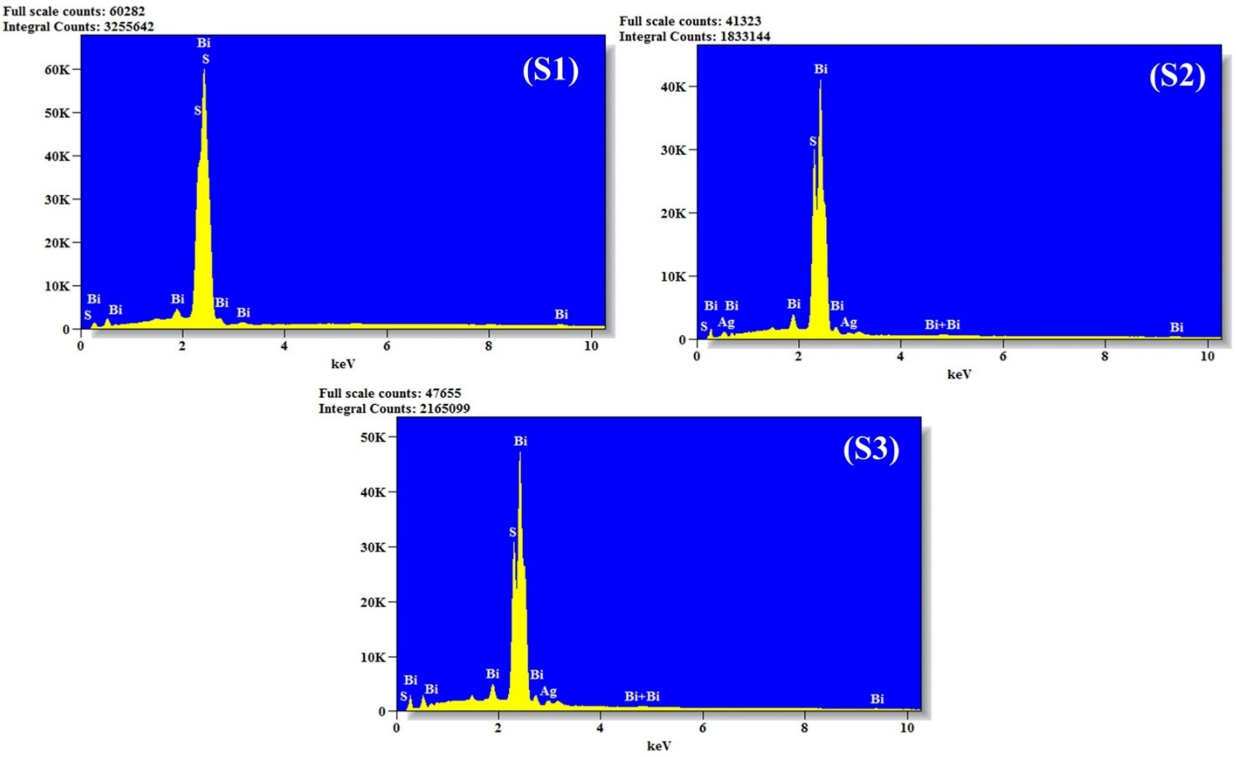

Fig. ES1 EDS spectra of Bi_2S_3 (S1), 0.01 % of Ag-doped Bi_2S_3 (S2) and 0.025 % Ag-doped Bi_2S_3 (S3) samples.

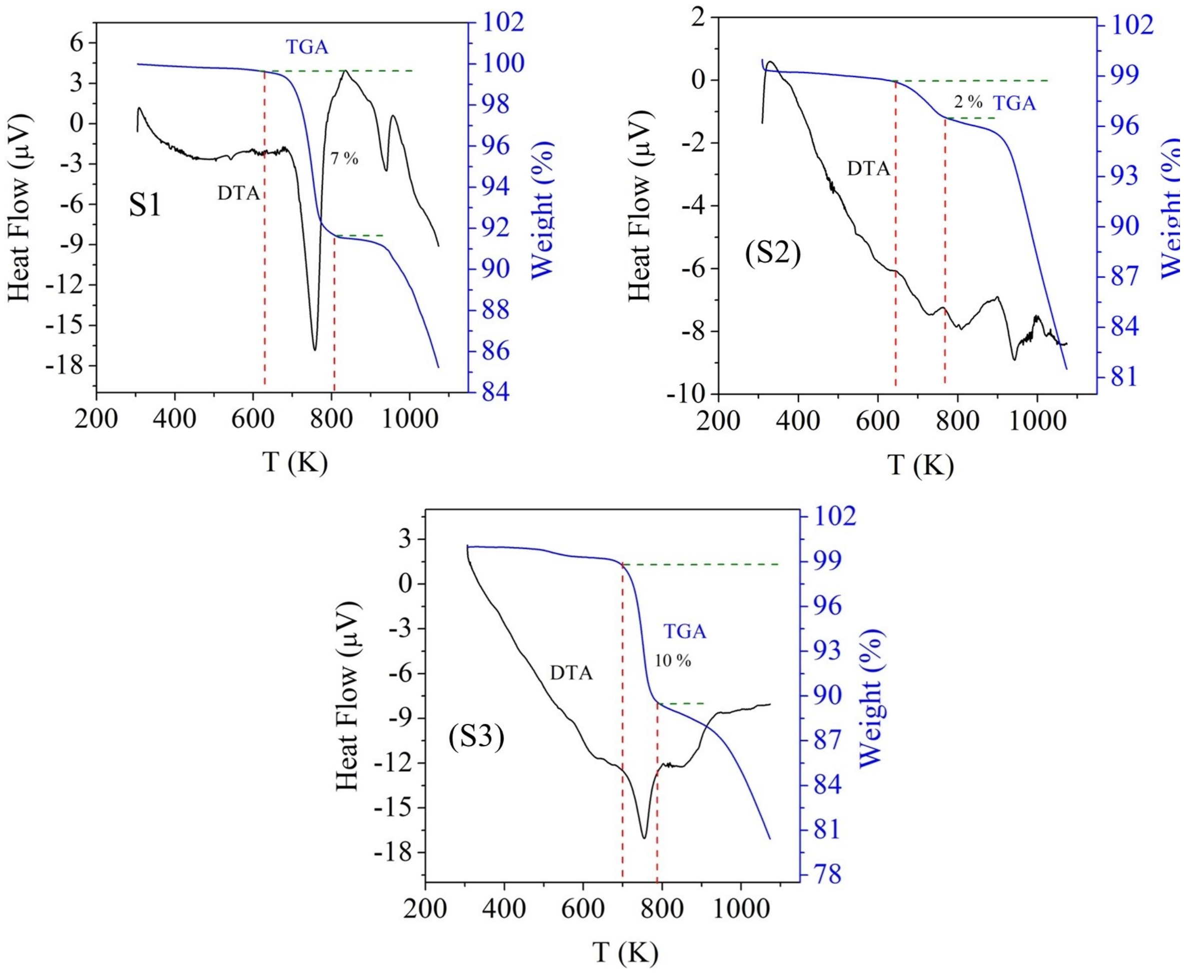

Fig. ES2: Thermogravimetric analysis (TGA) and differential thermal analysis (DTA) of Bi_2S_3 (S1), 0.01% Ag-doped Bi_2S_3 (S2), and 0.025% Ag-doped Bi_2S_3 (S3) samples.
